# UEG Journal—In Times of Turmoil, Science Matters

**DOI:** 10.1002/ueg2.70263

**Published:** 2026-07-09

**Authors:** Albrecht Neesse

**Affiliations:** ^1^ Gastroenterology, Gastrointestinal Oncology and Endocrinology University Medical Centre Goettingen Goettingen Germany

The past year was marked by the publication of many outstanding articles in UEG Journal spanning all areas of gastroenterology. These contributions have deepened our understanding of gastrointestinal diseases and will undoubtedly influence our clinical practice [1, 2, 3, 4, 5]. Selecting articles for this special issue was both a pleasure and a challenge, as I would have liked to include many more excellent papers from the past 12 months, but space is inevitably limited. In addition to original research and review articles [6], we published a record number of guidelines and consensus statements over the past year [7, 8, 9, 10]. We are proud to have become a premier venue for these interdisciplinary efforts. The Quality of Care committee at UEG continuously encourage authors to increase quality and standardization of guideline methodology to further raise the bar and provide the highest possible evidence and impact. In return, we actively promote all accepted articles and guidelines and enhance their visibility through social media, podcasts, and the many communication channels of UEG.

The international team of editors has developed a close and productive collaboration, not only during our in person meetings in Vienna and during UEG Week, but also through almost daily exchange. Our 14 trainee editors have gained considerable experience during their 2 years tenure with the journal, enabling them to take on increasing responsibilities with growing independence. In January 2027, a new international group of highly motivated and skilled trainees will join the UEG Journal. We received a record number of outstanding applications this year, reflecting the growing appeal of the programme, and I am looking forward to welcoming them to our team.

Professor Daniel Keszthelyi has been appointed Editor‐in‐Chief of *Neurogastroenterology & Motility*. Daniel served as Associate Editor of the UEG Journal from 2022 to 2025. We sincerely thank him for his outstanding work, expertise and dedication, and congratulate him on this well‐deserved appointment. At the same time, I am delighted to welcome Dr. Hans Törnblom, University of Gothenburg, Sweden, who joined our editorial team as Associate Editor for Neurogastroenterology in January 2026. Hans has already made a significant impact and has strengthened the team with his broad expertise [11, 12].

Maintaining the right balance between speed and quality in the peer‐review process is a daily challenge. We remain committed to continuously improving the quality, transparency and fairness of our editorial decisions as we select the most impactful papers for publication in the UEG Journal. Fortunately, submissions have increased once again this year, giving us an even stronger pool of high‐quality manuscripts from which to choose.

I hope you enjoy UEG Week, find time for both scientific discussion and personal interaction, and return home inspired by new ideas and fresh perspectives. At a time when the world is facing profound challenges and global conflicts, international collaboration, openness, mutual respect, and a commitment to diversity are more important than ever. UEG and UEG Journal provide an outstanding platform for fostering these principles beyond the borders of Europe. I also hope you enjoy reading this special issue, for which I selected excellent articles across the whole spectrum of gastroenterology including the best paper 2026 that was selected by our editorial team and will be awarded during UEG Week [13]. I also like to encourage you to listen to our UEG Journal podcasts, and follow us on social media.

Together, these shared efforts and activities give us confidence that we can overcome the many challenges—because in times of turmoil, science matters.

## Conflicts of Interest

The author declares no conflicts of interest.

## Data Availability

Data sharing not applicable to this article as no datasets were generated or analyzed during the current study.
